# Herpes Simplex Keratitis in Patients with SARS-CoV-2 Infection: A Series of Five Cases

**DOI:** 10.3390/medicina57050412

**Published:** 2021-04-24

**Authors:** Nora Majtanova, Petra Kriskova, Petra Keri, Zlatica Fellner, Juraj Majtan, Petr Kolar

**Affiliations:** 1 Department of Ophthalmology of Slovak Medical University and University Hospital in Bratislava, Antolska 11, 851 07 Bratislava, Slovakia; p.juhanesovicova@gmail.com (P.K.); petra.poropatichova@gmail.com (P.K.); zlaticafellner@gmail.com (Z.F.); petr.kolar@szu.sk (P.K.); 2 Faculty of Medicine, Slovak Medical University, Limbova 12, 833 03 Bratislava, Slovakia; 3 Institute of Molecular Biology, Slovak Academy of Sciences, Dubravska cesta 21, 845 51 Bratislava, Slovakia; juraj.majtan@savba.sk

**Keywords:** herpes simplex keratitis, COVID-19, immunosuppression, antiviral therapy

## Abstract

Herpes simplex virus type 1 (HSV-1) is a leading cause of infectious blindness worldwide. Most of the initial infection cases manifest as acute epithelial keratitis. Reactivation of herpesviruses is common in critically ill patients, including patients with severe Coronavirus disease (COVID-19). However, the data on COVID-19-related ocular infections is sparse, despite recent observations that more than 30% of COVID-19-infected patients had ocular manifestations. We report five cases of HSV-1 keratitis in COVID-19 patients. In total, five COVID-19 patients underwent ophthalmic examination, showing similar symptoms, including photophobia, tearing, decreased vision, eye redness, and pain. After initial assessment, tests of visual acuity and corneal sensitivity, a fluorescein staining test, and complete anterior and posterior segment examinations were performed. A diagnosis of HSV-1 keratitis was confirmed in all cases. Therapy was initiated using a local and systemic antiviral approach together with local antibiotic and mydriatic therapy. The complete reduction of keratitis symptoms and a clear cornea was achieved in all patients within 2 weeks. SARS-CoV-2 infection may be a risk factor for developing HSV-1 keratitis, or it may act as a potential activator of this ocular disease.

## 1. Introduction

Herpes simplex keratitis is characterised by recurrent infection of the cornea caused by herpes simplex virus type 1 (HSV-1), and less frequently by herpes simplex virus type 2 (HSV-2) [1]. In developing countries, it is the second leading cause of blindness after cataract surgery [1,2]. After primary infection, the virus is in its latent form in the nervous tissue (sensory and autonomic neurons of trigeminal and dorsal ganglia). Virus reactivation may occur in critically ill patients, or due to a weakened immune system, psychological stress, ultraviolet exposure, fever, or hormonal changes [3].

Severe acute respiratory syndrome coronavirus 2 (SARS-CoV-2) is an RNA beta coronavirus that causes coronavirus disease (COVID-19), first identified in Wuhan, the capitol of Hubei province. COVID-19 is an infectious disease that may cause not only SARS, but also the development of ocular symptoms. More than 30% of COVID-19 patients may have ocular manifestations [4,5].

The characteristics of the immune system in COVID-19 patients include immunosuppression and cytokine storm syndrome [6]. These characteristics are mostly found among critically ill patients. The number of T lymphocytes and natural killer cells is decreased, particularly CD8+ T cells. This immunosuppressive status may potentiate reactivation of latent viral infections such as HSV-1 [7].

Here, we report five cases of herpes simplex keratitis in COVID-19 patients, where the SARS-CoV-2 infection seems to be a potential activator.

## 2. Case Presentation

During the peak of the second pandemic wave in Slovakia (September 2020–January 2021), the total number of COVID-19 positive patients sharply increased in Bratislava region (Table 1). During this pandemic wave, a 2.5- and 2-fold higher incidence of herpes keratitis was also observed in our department in comparison to the same period in 2019 and 2018, respectively (Table 2). The prevalence of herpes keratitis was higher in male patients.

In 2018 and 2019 we diagnosed 11 and 9 patients with HSV-1 keratitis, respectively. There were no SARS-CoV-2 positive patients in these years. Between September 2020 and January 2021, 24 patients with HSV-1 keratitis were diagnosed. Eighteen of these patients were SARS-CoV-2 positive (Table 1). Further characteristics of these patients are described in Table S1.

### 2.1. Case 1

A 50-year-old woman presented to the emergency room at our department with a 2-week history of bilateral eye secretion associated with redness and 1-day history of left eye pain. She tested positive for SARS-CoV-2 using the RT-PCR test one week prior. The subject was myopic, but there was no history of wearing of contact lenses. No other health issues were stated.

Best corrected visual acuity in the right eye was 20/25, and 20/40 in the left eye. Slit lamp examination revealed conjunctival hyperemia in both eyes, with conjunctival secretion with a thin and seropurulent quality. The cornea of the right eye was intact, but in the left eye a typical HSV-1 dendritic lesion with the presence of fluorescent staining in the corneal centre was diagnosed. Topical antibiotic drops (levofloxacine 5 drops/day in both eyes) were added to her treatment, and topical acyclovir ointment (5 applications/day) and mydriatics (3 drops/day, homatropine) were prescribed for the left eye. Her treatment regimen also included oral acyclovir (400 mg, 5 tablets/day).

On follow-up five days later, the patient reported improvement in symptoms and visual acuity. Her visual acuity was 20/20 with no conjunctival secretion and no corneal staining in both eyes. The treatment was gradually reduced over the next two weeks with no recurrence of symptoms and signs. Additional follow up in 2 months was without signs of recurrence.

### 2.2. Case 2

In January 2021, a 71-year-old male patient was admitted into the emergency department for tearing, ocular pain, photophobia, and worsened vision in the right eye. The ophthalmological exam revealed a visual acuity of 20/50 in the right eye and counting fingers in the left amblyopic eye. Intraocular pressure values were normal. At slit lamp examination, the patient was diagnosed with conjunctival hyperemia, paracentral epithelial defect (staining with fluorescein), corneal subepithelial haze, and anterior chamber inflammation (tyndalisation ++). Corneal sensation was significantly reduced. The patient history revealed herpes simplex keratitis in the right eye in May 2020.

Based on the symptoms and the ophthalmological examination, the patient was diagnosed with recurrent herpes simplex keratitis with anterior chamber inflammatory activity in the right eye. The patient tested positive for SARS-CoV-2 by RT-PCR testing on the same day.

Topical therapy was started in the right eye with levofloxacine (5 drops/day), mydriatics (3 drops/day, homatropine), and acyclovir ointment (5 applications/day). Systemic therapy was initiated with intravenous acyclovir (5 mg/kg, 3 times/day) for an initial 5 days.

The evolution under this treatment was good. The patient was monitored twice a day at our department. At slit lamp examination the epithelial defect was smaller and subepithelial haze was reduced in the right eye. After five days, the intravenous acyclovir was changed to oral acyclovir (400 mg, 5 tablets/day).

On follow up two weeks later, the patient’s visual acuity in the right eye was improved to 20/25, conjunctival hyperemia was significantly reduced, and the epithelial defect with subepithelial haze had disappeared. Topical and systemic therapy were gradually discontinued.

On the last examination 2 months later, there were no signs of recurrent HSV-1 infection in the right eye. Visual acuity had remained stable at 20/25 and intraocular pressure was at physiological values. The anterior chamber was free from inflammatory activity.

### 2.3. Case 3

A 43-year-old woman had tested positive for SARS-CoV-2 by RT-PCR three days before she came to our department with itching and burning in the right eye. In childhood, she underwent strabismus surgery in the right eye and stayed aphakic after surgery for congenital cataracts in both eyes. She had been regularly monitored for serous retinal detachment in the right eye with light perception.

Upon examination, her visual acuity was light perception in the right eye and 20/20 with hypermetropic aphakic correction in the left eye. Slit lamp examination of the right eye revealed conjunctival hyperemia and excretion, a dendritic epithelial defect directly under the centre of the cornea (1.5 mm with fluorescein staining), descement membrane folds, and diffusely decreased corneal transparency. There was no corneal sensation in the right eye. The patient was diagnosed with herpes simplex keratitis in the right eye. Initial topical treatment in the right eye was with levofloxacine (5 drops/day), mydriatics (3 drops/day, homatropine), and acyclovir ointment (5 applications/day). Systemic therapy included oral acyclovir (400 mg, 5 tablets/day).

On follow-up four days later, conjunctival injection was reduced, mild descemet membrane folds persisted, and the cornea became more transparent with a reduced epithelial defect.

A month later, the evolution under treatment was good and the cornea was completely cured. Visual acuity in the right eye remained light perception due to pre-existing serous retinal detachment. The cornea was completely healed with subepithelial haze in the region of primary HSV-1 keratitis.

### 2.4. Case 4

A 49-year-old woman was referred to our department with a 2-week history of pain associated with photophobia, conjunctival secretion, and blurred vision in both eyes. Her ocular history was significant, with primary open-angle glaucoma and bilateral allergic conjunctivitis. The patient did not tolerate local antiglaucomatics due to an allergic reaction.

Prior to the presentation, the patient was unsuccessfully treated for decompensated primary open-angle glaucoma with worsening keratitis in both eyes for two weeks. Intraocular pressure was high, between 40 and 50 mmHg. The patient’s topical treatment was tobramycin-dexamethasone (5 drops/day), autologous serum from blood (5 drops/day), and anti-glaucoma drops (latanoprost-timolol and brinzolamide-brimonidine). Systemic treatment consisted of acetazolamide (3 tablets/day).

On the day of presentation, the ophthalmological exam revealed visual acuity in the right eye was 20/25 and in the left eye was 20/32, with raised intraocular pressure between 50 and 60 mmHg in both eyes. At slit lamp examination, the patient had conjunctival hyperaemia with seropurulent secretion, corneal oedema, and reduced corneal sensitivity in both eyes. In the right eye, a paracentral linear epithelial defect with fluorescein staining was observed. In the left eye, below the corneal centre, a dendritic epithelial defect was also present. Due to reduced corneal transparency, an ultrasound was used to exclude pathological changes in the posterior segment. Ultrasound of the right eye showed mild vitritis. Based on the clinical changes, the patient was diagnosed with bilateral acute keratoconjunctivitis (HSV-1 suspect) and decompensated secondary glaucoma with vitritis in the right eye.

On the same day, the patient tested positive for SARS-CoV-2 by RT-PCR and a new therapy regimen was started. Topical therapy in both eyes included levofloxacine (5 drops/day), dexamethasone (5 drops/day), mydriatics (3 drops/day, homatropine), acyclovir ointment (5 applications/day), and artificial tear drops. Intravenous acyclovir (5 mg/kg, every 8 h), cefotaxime (2 g/day), and mannitol (10% 250 mL/day) were added to the systemic therapy. Cefotaxime therapy was added due to the vitritis, that was diagnosed in the right eye.

The patient was checked twice a day at our department. Five days later, the patient reported decreased pain and improved vision. The evolution under treatment was successful. Intraocular pressure returned to normal values, visual acuity in both eyes improved to 20/20, and the keratitis in both eyes and vitritis in the right eye diminished. After one week, systemic antiviral and antibiotic therapy was changed to oral therapy, and intravenous mannitol was stopped.

Two weeks later, the patient came to follow-up. She felt very well and had no complaints about her condition. Biomicroscopic examination showed minimal conjunctival hyperemia, with punctate corneal epithelopathy with dot-like staining of the corneal epithelium. Systemic therapy was terminated, and topical therapy was changed to preservative-free local anti-glaucoma therapy (latanoprost and dorzolamide hydrochloride-timolol maleate) and artificial tear drops.

On follow-up one month later, there was additional biomicroscopic improvement of both eyes, with visual acuity of 20/20 and normal intraocular pressure values of 20 mm Hg.

### 2.5. Case 5

**Figure 1:**
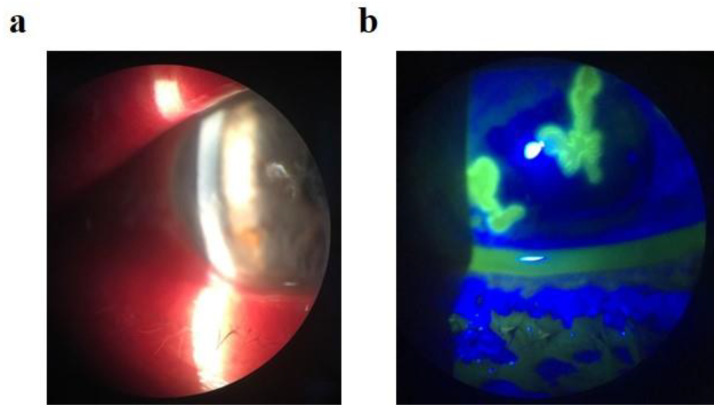
A representative image of herpetic keratitis in the five described cases. (**a**) Slit-lamp examination with herpetic lesion. (**b**) Slit-lamp examination with fluorescein dye and blue light, showing typical corneal dendritic lesions.

A 27-year-old physician, who had tested positive for SARS-CoV-2 by RT-PCR 9 days prior, presented to the emergency ophthalmology department. He complained about mild eyelid swelling with redness, foreign body sensation, and photophobia in both eyes. In the ocular history of this patient was recurrent bilateral herpetic keratitis in childhood and adulthood. At the time of presentation, his visual acuity was 20/20, with normal intraocular pressure in both eyes. Biomicroscopic examination revealed a blistery rash on the skin of the eyelids (more dominantly on the left site), bilateral conjunctival hyperemia with mucous excretion, and dendritic corneal epithelial defects with reduced corneal sensitivity in both eyes. Based on the ophthalmological examination, the patient was diagnosed with bilateral Herpes simplex blepharitis and keratoconjunctivitis (Figure 1). Topical levofloxacine (5 drops/day), mydriatics (3 drops/day, homatropine), and acyclovir ointment (5 applications/day) were added to his treatment for both eyes. Systemic therapy included oral acyclovir (400 mg, 5 tablets/day).

On follow-up three days later, the patient reported an improvement in symptoms. His visual acuity was 20/20, with reduction the blistery skin rash, no conjunctival excretion, and minimal staining of dendritic corneal defects in both eyes. Two weeks later, all herpetic lesions were completely healed.

Two months later the patient reported no complaints. Visual acuity was 20/20 in both eyes. There was very mild corneal haze, which did not interfere with the visual axis. The patient was satisfied with result of the treatment.

## 3. Discussion

HSV is transmitted throughout the world. According to some studies, the latent form of HSV-1 infection was diagnosed in 90% of world population older than 60 [8]. HSV-1 seroprevalence appears to be decreasing, with an increasing number of HSV-2 cases [9]. After oral herpetic infection, the second most common localisation of symptomatic HSV-1 infection is the eye. The most frequently affected area is the cornea (keratitis), but it may also manifest as conjunctivitis, uveitis, or acute retinal necrosis [9]. Weakening of the immune system and the presence of inflammatory mediators such as cytokines are important factors in the reactivation of HSV-1 infection [10].

There is little data about the incidence of ocular HSV. To our knowledge, there are only a few studies that have estimated ocular HSV incidence. In 1970 and 1979, two such studies were published in Denmark, three papers were published in the United States (1950–2007), and other studies originated from Croatia, the United Kingdom, and France. According to all of these studies, the incidence of new cases of HSV eye disease is between 5 and 15 per 100,000 population per year. It is estimated that there are 1,000,000 new cases of the ocular form of HSV and 9,000,000 recurrences of this infection per year [8].

Between 01 September 2020 and 31 January 2021, the incidence of COVID-19 patients rose sharply in Slovakia [11]. During this period, we examined 24 patients with ocular HSV-1 infection at our department. Six of them had no history of COVID-19 and a negative test for SARS-CoV-2 antibodies (IgM and IgG). Surprisingly, 18 of these patients had either a positive RT-PCR test for SARS-CoV-2 or tested positive for SARS-CoV-2 antibodies. Seventy-five percent of patients from this group affected by the ocular form of HSV-1 overcame their COVID-19 disease.

The characterisation of the immune system in COVID-19 patients shows a decreased number and functional exhaustion of natural killer (NK) cells, and cytotoxic T-lymphocytes [12]. These cells are responsible for the control of viral infection. According to a cohort study led by Zheng et al. in China, a significant decrease in NK cells and CD8+ T cells was found in both mild and severe COVID-19 cases. Expression of NKG2A, an inhibitory receptor, is significantly increased on NK cells and CD8+ T cells in COVID-19 patients [12], leading to their functional exhausting. There is also found a decreased percentage in IFN-γ+ NK, IL-2+ NK, CD107a+ NK, TNF-α+ NK cells, IL-2+ CD8+, IFN-γ+ CD8+, CD107a+ CD8+ T cells, and MFI of granzyme B+CD8+ T cells [12]. Thus, it seems that SARS-CoV-2 may cause a failure of antiviral immunity [12].

HSV-1 in its latent form resides in the trigeminal ganglion. This latent form is maintained by HSV-1-specific CD8+ T cells. The exhaustion of these cells leads to impaired effector function and memory cell loss, which potentiates reactivation of the virus [13]. In accordance with these mechanisms, COVID-19 may be a potential activator of HSV-1 infection. On the other hand, acyclovir could be used as a potential add-on treatment to the COVID-19 treatment regimen [14].

We saw and diagnosed an increased number of subjects with HSV-1 keratitis in the colder autumn and winter months (September-January), consistent with authors that published seasonal differences in HSV-1 occurrence [15,16]. These authors anticipated that HSV-1 infection recurrence is more common during the cold months due to a decrease in outside temperature. Other authors reported an increase in acute retinal necrosis caused by HSV-1 in the winter period [17]. It seems that outside temperature is very important factor in the recurrence of HSV-1 infections.

## 4. Conclusions

In summary, we report five cases of herpes simplex keratitis in COVID-19 patients. SARS-CoV-2 infection may be a risk factor for developing HSV-1 keratitis, or it may act as a potential activator of this ocular disease.

## Figures and Tables

**Table 1:** The increase in subjects testing positive for SARS-CoV-2 in the Bratislava region (Slovakia).

Time Period		Total Cases
**01-September-2018–31-January-2019**		0
**01-September-2019–31-January-2020**		0
**01-September-2020–31-January-2021**		19,631
	September 2020 October 2020 November 2020 December 2020 January 2021	951 2668 2620 6881 6511

**Table 2:** Prevalence of herpes simplex virus-1 in the Department of Ophthalmology (Slovak Medical University, Bratislava, Slovakia), between September 2020 and January 2021. Eighteen of 24 patients were SARS-CoV-2 positive.

Factor/Time Period		01-September-2018 31-January-2019	01-September-2019 31-January-2020	01-September-2020 31-January-2021 Total (SARS-CoV-2 Positive)
Total cases		11	9	24 (18)
Gender	Male Female	7 4	5 4	15 (11) 9 (7)
Age range (year)	18–29 30–39 40–49 ≥50	1 3 3 4	2 0 3 4	4 (3) 6 (4) 5 (4) 9 (7)

## Data Availability

The data presented in this study are available on request from the corresponding author. The data are not publicly available due to patients’ privacy.

## Supplementary Materials

The following are available online at https://www.mdpi.com/xxx, Table S1: The ophthalmic characteristics of patients positive for SARS-CoV-2 infection and diagnosed with herpes simplex virus-1 keratitis.
