# Supplementary material for: Herpes Simplex Keratitis in Patients with SARS-CoV-2 Infection: A Series of Five Cases
Source: Medicina (Kaunas). 2021 Apr 24;57(5):412. doi: 10.3390/medicina57050412 (PMC8146587; doi:10.3390/medicina57050412)
Supplement: Supplementary file 1 [file medicina-57-00412-s001.zip › medicina-1161829-supplementary.pdf]

Table S1. The ophthalmic characteristics of patients positive for SARS-CoV-2 infection and diagnosed with herpes simplex virus-1 keratitis.

Factor			Total cases
**The patients positive for SARS-CoV-2 by RT-PCR with herpes simplex-1 keratitis**			**18**
**BCVA of the affected eye**	Before treatment	20/20 - 20/25	4
		20/32 - 20/50	6
		20/63 - 20/100	3
		20/125 - 20/200	3
		Counting fingers	1
		Hand motion	0
		Light perception	1
		No light perception	0
	After treatment	20/20 - 20/25	12
		20/32 - 20/50	3
		20/63 - 20/100	1
		20/125 - 20/200	1
		Counting fingers	0
		Hand motion	0
		Light perception	1
		No light perception	0
**Length of treatment**		< 7 days	0
		8-14 days	2
		15-21 days	5
		22- 28 days	8
		> 28 days	3
**COVID-19 RT-PCR test**	Prior to HSV-1 keratitis diagnosis	> 14 days	2
		14 - 8 days	3
		7 - 3 days	4
		2- 0 days	7
		1-2 days	2

Factor			Total cases
	After HSV-1 keratitis diagnosis	3 - 7 days	0
		7 - 14 days	0
		> 14 days	0

Note: BCVA, best-corrected visual acuity; HSV-1, herpes simplex virus-1
